# Editorial: Biosurfactants - next-generation biomolecules for enhanced biodegradation of organic pollutants, volume II

**DOI:** 10.3389/fmicb.2024.1513087

**Published:** 2024-11-12

**Authors:** Punniyakotti Parthipan, Liang Cheng, Aruliah Rajasekar, Obulisamy Parthiba Karthikeyan, Pattanathu K. S. M. Rahman

**Affiliations:** ^1^Department of Biotechnology, Faculty of Science and Humanities, SRM Institute of Science and Technology, Chengalpattu, Tamil Nadu, India; ^2^School of Environment and Safety Engineering, Jiangsu University, Zhenjiang, China; ^3^Environmental Molecular Microbiology Research Laboratory, Department of Biotechnology, Thiruvalluvar University, Vellore, Tamil Nadu, India; ^4^Department of Prothodontics, Adjunct Faculty, Saveetha Dental College and Hospital, Chennai, Tamil Nadu, India; ^5^Department of Civil and Environmental Engineering, South Dakota Mines, Rapid City, SD, United States; ^6^Austin Elements Incorporation, Houston, TX, United States; ^7^Centre for Natural Products Discovery, School of Pharmacy and Biomolecular Sciences, Liverpool John Moores University, Liverpool, United Kingdom

**Keywords:** compound lipids, bioavailability, surface active molecules, optimization, hydrophobic pollutants, surface interaction

Organic pollutants such as hydrocarbons, microplastics, polyaromatic hydrocarbons (PAHs), and organochlorine pesticides are highly toxic to the environment and human health. Mostly these pollutants originate from anthropogenic activities (oil spills, burning of wood, extreme usage and dumbing of plastics, and industrial and agricultural activities) and sometimes from natural causes (volcanic activity, forest fire, and natural oil seeps). The removal of these organic pollutants from a contaminated environment is very challenging due to their extreme hydrophobic characteristics, which lead to persistence in the environment for many years (Elumalai et al., 2021, 2024b). Due to these properties and their fate, organic pollutants are easily transported to the different levels of the food chain and finally accumulate in the internal organs of the animals, particularly in humans. The prolonged accumulation of some pollutants, such as PAHs, in the human tissues is highly dangerous since they are recognized as carcinogenic and mutagenic with endocrine disruption abilities (Phulpoto et al., 2024).

There are several physicochemical methods (adsorption, advanced oxidation, burning (for crude oil spills in oceans), coagulation, flocculation, electrochemical oxidation, filtration, etc.) that have been applied to minimize or remove these hazardous hydrophobic organic pollutants from environments. Most of these physicochemical approaches are effective for the removal but end up with some toxic byproducts that cause secondary pollution to the various ecosystems (Elumalai et al., 2024a). Bioremediation is an alternative, eco-friendly, and sustainable approach to removing these persistent pollutants from different environmental conditions either by *in-situ* (directly at the site of contamination) or *ex-situ* (contaminated soil transported to another location) methods. The most challenging part in the removal of these organic pollutants is their hydrophobic nature, which decreases their availability to the microorganisms (Bose et al., 2024). This bioavailability can be enhanced by applying surfactants, which form micelles with the hydrocarbons and reduce the surface and interface tension, which helps for the efficient biodegradation process (Pardhi et al., 2022). Most of the synthetic surfactants are causing numerous environmental impacts, so they need to be replaced by the biosurfactant eco-friendly surface-active molecules.

The diverse range of microorganisms producing different types of these amphipathic molecules belongs to the compound lipids (glycolipids, phospholipids, and rhamnolipids) and lipoproteins (surfactin and iturin). Biosurfactants have plentiful compensations over chemical surfactants, including less toxicity, biodegradability, and stability in extreme environmental conditions. Different types of biosurfactants are used in many industrial applications, such as the food industry, cosmetics, detergents, nanomaterial synthesis, pharmaceuticals, oil recovery, and bioremediation (Walaszczyk et al., 2023; Sankhyan et al., 2024). In addition, biosurfactants can be produced using easily available substrates, including agro-wastes and industrial waste, which favors cost-effective large-scale production, thus also minimizing industrial and agricultural waste disposal and associated environmental problems (Markam et al., 2024).

As mentioned earlier, biosurfactants are a very effective candidate that can make an impact on the sustainable bioremediation process efficiently due to their extraordinary compatibility and nature (Ng et al., 2022). In the bioremediation process, especially at the large-scale field conditions, till now very few times only applied with significant outcomes. Accidentally, many times hydrocarbon contaminations occur frequently during transport, but there is no immediate solution available even though such surface-active materials are proven as options to treat them. This might be due to the fewer field studies carried out with different types of biosurfactants, and most of the studies are limited within the lab conditions, lacking potential strains or potential strains are not preserved for longer use (Pritchard and Costa, 1991). This Research Topic is volume II of the same concept, which is biosurfactant-based biodegradation of organic pollutants. The focus of this Research Topic was to update the recent trends in the bioremediation applications, which included biosurfactants for the enhanced removal of various hydrophobic pollutants.

The study “*Enhancement of cell migration and wound healing by nano-herb ointment formulated with biosurfactant, silver nanoparticles and Tridax procumbens*” by Muthukumar et al. adapted the critical micelle forming ability of the rhamnolipid biosurfactant produced by a potential biosurfactant producing bacterial strain, *Pseudomonas aeruginosa* PP4. In this study, biosurfactants are used as emulsifying agents in the formulations of the wound healing nano-herb ointment. Due to the inclusion of the rhamnolipid biosurfactant, the formulated ointment exhibited its stability for a longer duration without losing its functional properties. Overall, due to the inclusion of the eco-friendly biosurfactant in this nano-herb ointment preparation, low cytotoxic and higher cell migration capacity are obtained, which are very crucial factors for effective wound healing applications. This study also revealed that this kind of potential strain can be adapted for environmental remediation applications as well.

A mini-review paper published on this Research Topic, “*Biosurfactant: an emerging tool for the petroleum industries*,” by Sharma et al. summarized the major applications of the biosurfactants in the petroleum industry as microbial-enhanced oil recovery process, applications in heavy viscous crude oil transport and storage tank cleaning purposes, and how they are used in oil spill treatment. In addition, possible other applications include how utilized as a corrosion inhibitor to suppress microbial corrosion in the oil industry, especially against the sulfate-reducing bacterial group. Similarly, another review “*Advancements in biosurfactant production using agro-industrial waste for industrial and environmental applications*,” by Sundaram et al. summarized the major advantages of biosurfactants for environmental applications. In addition, this review focused on the low-cost production strategies of the biosurfactant by adapting an effective fermentation approach with easily available waste substrate.

A novel strain with its optimum condition could help microorganisms produce their secondary metabolites with the maximum capacity. In a study, “*Production and characterization of rhamnolipid biosurfactant from thermophilic Geobacillus stearothermophilus bacterium isolated from Uhud mountain*,” Albasri et al. isolated a thermophilic bacterium from the volcanic and arid region of Uhud mountain, Madinah, Saudi Arabia. In this study, waste sunflower frying oil has been used as one of the effective carbon sources to produce a higher amount of biosurfactant. This revealed that biosurfactants can be produced on a larger scale using agricultural waste material. In addition, there are other possible applications such as antimicrobials, antioxidants, and plant growth-promoting agents.

Crude oil contamination under various environmental conditions is a very common problem due to the widespread exploration and transport of crude oil from offshore locations. Bioremediation is one of the safer and more effective approaches for the management of crude oil contamination. In a study “*Molecular identification of rhamnolipids produced by Pseudomonas oryzihabitans during biodegradation of crude oil*,” Hosseini et al. isolated a rhamnolipid-producing bacterial strain, *Pseudomonas oryzihabitans* NC392, and it exhibited a great critical micelle concentration with 33 mN m^−1^ at 200 mg L^−1^ concentration and found that this biosurfactant could be a potential candidate to remediate the hydrophobic organic pollutants such as for the crude oil and also can be used for the microbial enhanced oil recovery.

As mentioned earlier, hydrophobic contaminants removal is a very challenging task due to their unavailability. To tackle this, a study “*Solubilization and enhanced degradation of benzene phenolic derivatives-bisphenol A/triclosan using a biosurfactant producing white rot fungus Hypocrea lixii S5 with plant growth promoting traits*” by Chaturvedi et al. produced a surfactin type of biosurfactant with *Hypocrea lixii*, a white rot fungal strain. This strain enhanced the degradative enzyme production and played a crucial role in promoting plant growth-supporting components. If this type of biosurfactant is adapted for the integrated remediation approach such as phytoremediation, the added biosurfactant could help to restore the contaminated environment.

Overall, these six different studies determined the efficacy and importance of biosurfactants in the bioremediation of hydrophobic organic pollutants. The outcomes of this Research Topic undoubtedly specified the need for biosurfactants for environmental and industrial applications. In-depth studies would be helpful for the improvement of biosurfactant screening and production for large-scale environmental applications.
